# Editorial to the thematic series 'Invertebrate Circuitry'

**DOI:** 10.1186/2042-1001-1-10

**Published:** 2011-07-26

**Authors:** George Kemenes

**Affiliations:** 1 School of Life Sciences, University of Sussex, Falmer, Brighton, BN1 9QG, UK

## 

As a member of the Editorial Board who works in the field of invertebrate neuroscience, I feel very privileged by being able to introduce here the first thematic series of *Neural Systems & Circuits *[1]. Befitting the evolutionary history of neuronal circuits in the animal kingdom, this first thematic series will be entitled 'Invertebrate Circuitry'. However, it is not only the evolutionary 'seniority' of invertebrates that justifies the dedication of this series to invertebrate neural systems and circuits. It is well known these days that work on invertebrate neurons and circuits has made a key contribution to our understanding of many fundamental processes governing the function of all nervous systems, including the human brain. These evolutionarily highly conserved processes include the ionic mechanisms of the membrane potential and action potential of nerve cells, the generation of receptor potentials, the one-way conduction of chemical synapses, the role of electrical synapses and the basic physiological mechanisms of the visual response in the retina, to name but a few. Importantly, invertebrate systems have also provided the first detailed mechanistic insights into the more complex functions of neuronal circuits, such as central pattern generation, decision making, neuromodulation, synaptic plasticity and learning and memory, and the development of neuronal networks.

Ramón y Cajal wrote it most eloquently in the first chapter of *Histology of the Nervous System *[2]: '...one can learn a great deal about the morphology, connections, and fine structure of nerve cells by carefully studying those animals in which nervous systems first evolved...'. More than a hundred years later the only thing we can add to this is that at an even more general level, the most important lesson that we can learn from past and current (and hopefully, future) invertebrate studies is how individual neurons interact to generate specific kinds of behaviour.

In this thematic series we expect to publish both reviews and original studies on invertebrate model systems (including worms, crustaceans, insects and molluscs) in which any aspects of circuit function are currently being investigated, using either experimental or computational approaches. The behaviours whose underlying circuitry will be described in the series include simple defensive reflexes, complex rhythmic behaviours, such as swimming and crawling in the leech, insect flight, feeding in molluscs and even more complex behaviours, such as phase change in locusts or path integration and spatial navigation in ants. The powerful combinations of anatomical, genetic, behavioural, physiological and computational methods that can be applied in invertebrate systems are aiding our understanding of circuitry in general by elucidating how intrinsic neuronal properties, synaptic connectivity and network organization together contribute to systems level circuit function and the generation of a variety of well-defined behaviours.

We have already commissioned a number of reviews from leading experts in invertebrate circuitries (the first two are published in this issue) but I would also like to use this opportunity to call for new and exciting research to add to the series.
